# Case of Opisthotonos, Successfully Treated

**Published:** 1803-12-01

**Authors:** R. Nayler

**Affiliations:** Surgeon to the Gloucester Infirmary


### CASE of OPISTHOTONOS, *successfully treated, by* R. NAYLER, *Surgeon to the Gloucester Infirmary.*

THOMAS ORGAN, 36 years of age, in the parish of Witcomb, about five miles distant from Gloucester, in his usual employ as a husbandman, in the month of February, 1802, had his left hand severely crushed, the second joint of his middle finger much lacerated, and the bone fractured. On receiving the accident, he immediately set off for my house, where, in my absence, the necessary care was taken of him by a young gentleman, then my apprentice. From this time every thing went on as kindly as could be wished, and in the course of a month he was so far recovered with an anchylosis of the injured joint as to enter upon some of the less laborious parts of his employment. The weather unfortunately for him was at this time very cold and moist, which affected him so much that he returned home early in the evening greatly fatigued and much indisposed. He passed the succeeding night very uncomfortably, distressed by wandering pains in his shoulders and back, and feeling the usual symptoms of what is called a cold. Unwilling however to yield at once, he made another effort the next morning, but soon left his work under an aggravation of all his complaints. He was now bled, took a strong sweating medicine without any relief, he therefore lost more blood, but without the least diminution of his sufferings. In this state I saw him on the third day after his attack, and then found him under a confirmed locked jaw.

The muscles of the neck and shoulders every now and then suffered the most irregular convulsions, succeeded by such rigid contractions of those of the back, as to draw his head forcibly backwards at the same that his chest was considerably elevated, and through which a very acute pain frequently darted to his back. His countenance was frightfully distorted, and bore the strongest possible marks of the agony he suffered. His pulse was about 80 in a minute. He had no preternatural heat, thirst, or indeed any symptom of fever, nor had he felt the least pain in the injured finger. Bladders of warm water were ordered to his feet, and forty drops of laudanum to be taken every four hours in a camphorated mixture with tincture of castor. His bowels to be kept open by castor oil, calomel, glysters, &c. and blisters were applied on his chest, and also on the cicatrix of the injured finger. These means were were made use of several days without any material reduction of symptoms, though, to give a full trial to what opium could do, there was an increase of twenty drops of laudanum to each dose, given as before, every four hours. At my next visit I found him much more irritable, and that every time he attempted to speak or swallow, he became more convulsed, with an apparent aggravation of all his very distressing symptoms. The muscular rigidity had now extended itself to his lower extremities, and, though in a less degree, to his arms also. His pulse was become quick and small, his face occasionally flushed, and he perspired excessively; whilst there was an appearance of stupidity which I could not but attribute more to the imperfect action of the opiate, than any affection of the head. He was now ordered wine freely, to the amount of a bottle in the day, with a strong decoction of the bark and valerian, at the same time a liniment well charged with opium was rubbed into his chest and limbs twice a day, but without producing the least relaxation in the violence or frequency of the spasms. The rigidity was now become universal, except with regard to his fingers, over which he had a trifling degree of power.

In this most deplorable state he reached the fourteenth day from the attack, under the impression on all about him that every paroxysm would take him off; when visiting once more, the application of cold water occurred to me, as having been tried under similar circumstances; and therefore, having combated for some time the prejudices of his surrounding friends against its use, I at last prevailed on them to remove him out of bed, and allowing him as much wine as he could take, (about a tea cup full) whilst supported between two persons, to dash about a quart of cold water over his face, chest, and back, and to repeat the same every four hours, increasing both the force and quantity of the water at each succeeding operation, as he appeared equal to it. The first experiment gave him so much and immediate relief, that he looked forwards with no small anxiety to a repetition of it at the appointed periods; and pursuing it to the twenty-seventh affusion, his spasms had entirely left him, those affecting his jaw first giving way, and successively those of his chest, back, and extremities.

To recover him from the very debilitated state to which he was reduced, the bark and a cordial diet were continued, till by a gradual recovery he was able to return to his usual usual occupation, which he now follows without any remaining inconvenience whatever.

It is worthy of remark, that on being taken out of bed to receive the first affusion of cold water, he appeared like a corpse, or one frozen to death. So rigid and inflexible were all his joints, and his feet so pointedly stiffened, that when placed between the assistants to sustain the operation, he rested on the extremities of his toes only. It is likewise to be observed, that the large doses of opium joined with the antispasmodic remedies were of no avail, even as palliatives, and that there is not the least ground to conclude that the disease was subdued by the efforts of the constitution. The amendment began with the first external application of cold water, it became progressive as the cold affusions were more frequently and effectually applied, and a complete cure was evidently the result of their continuance. That the cold bath has been employed in vain in similar cases, is a fact known to medical men. Are we then to attribute our success in this case to the *less violent*, and mere *local* application of cold? or, if not to these, to what other circumstance are we to impute this favourable event?

